# Evidence of a reduction in cloud condensation nuclei activity of water-soluble aerosols caused by biogenic emissions in a cool-temperate forest

**DOI:** 10.1038/s41598-017-08112-9

**Published:** 2017-08-16

**Authors:** Astrid Müller, Yuzo Miyazaki, Eri Tachibana, Kimitaka Kawamura, Tsutom Hiura

**Affiliations:** 1 Institute of Low Temperature Science, Hokkaido University, Sapporo, 060-0819 Japan; 2 Graduate School of Environmental Science, Hokkaido University, Sapporo, 060-0810 Japan; 3 Chubu Institute for Advanced Studies, Chubu University, Kasugai, 487-8501 Japan; 4 Field Science Center for Northern Biosphere, Hokkaido University, Tomakomai, 053-0035 Japan

## Abstract

Biogenic organic aerosols can affect cloud condensation nuclei (CCN) properties, and subsequently impact climate change. Large uncertainties exist in how the difference in the types of terrestrial biogenic sources and the abundance of organics relative to sulfate affect CCN properties. For the submicron water-soluble aerosols collected for two years in a cool-temperate forest in northern Japan, we show that the hygroscopicity parameter κ_CCN_ (0.44 ± 0.07) exhibited a distinct seasonal trend with a minimum in autumn (κ_CCN_ = 0.32–0.37); these κ_CCN_ values were generally larger than that of ambient particles, including water-insoluble fractions. The temporal variability of κ_CCN_ was controlled by the water-soluble organic matter (WSOM)-to-sulfate ratio (R^2^ > 0.60), where the significant reduction of κ_CCN_ in autumn was linked to the increased WSOM/sulfate ratio. Positive matrix factorization analysis indicates that α-pinene-derived secondary organic aerosol (SOA) substantially contributed to the WSOM mass (~75%) in autumn, the majority of which was attributable to emissions from litter/soil microbial activity near the forest floor. These findings suggest that WSOM, most likely α-pinene SOA, originated from the forest floor can significantly suppress the aerosol CCN activity in cool-temperate forests, which have implications for predicting climate effects by changes in biogenic emissions in future.

## Introduction

Atmospheric aerosols play a key role in the climate system as they act as cloud condensation nuclei (CCN), and impact cloud formation^1^. The major factors controlling the activation of CCN include particle solubility and hygroscopicity, which are functions of chemical composition^2, 3^. The hygroscopicity parameter κ describes the influence of chemical composition on the CCN activity of aerosol particles^4^. Sulfate aerosol is one of the most effective components of CCN^5^ with κ values of greater than 0.6, whereas organics generally have lower κ values^6^, typically below 0.3 for biogenic secondary organic aerosols (BSOAs), for example. In forested regions with strong influence of biogenic sources, organic matter (OM) accounts for substantial fractions of the submicron particle mass. A majority of biogenic OM in submicron particles consists of BSOA derived from biogenic volatile organic compounds (BVOCs) including isoprene and monoterpenes, whereas primary biological aerosol particles (PBAPs) can also be a significant source of organic aerosol (OA). Because a large fraction of this biogenic OA is water-soluble^7^, the formation and abundance of biogenic OA relative to sulfate can modify CCN activity when organics coat sulfate particles. This type of coating can change the surface composition and water affinity of the particle.

Previous field studies conducted in areas dominated by biogenic OA, such as the Amazon^8, 9^, boreal forests^10–12^ or mountainous forests^13, 14^, have reported κ values below 0.3. Some studies have reported higher hygroscopicity for particles with larger diameters between 0.1 and 2.5 µm, which can be explained by the enrichment of sulfate in those particle size ranges^9, 14, 15^. Meanwhile, lower κ values for particles with diameters between 10 and 100 nm have been attributed to a larger fraction of organic mass^9, 14, 15^. Because most of the κ measurements were made by intensive field campaigns for periods of a few weeks, it is worth investigating how κ changes relative to changes in biogenic emissions throughout the year. Seasonal variability in CCN concentrations and aerosol hygroscopicity has been previously investigated in forested areas^11–13, 16, 17^, although limited chemical data is available to fully interpret them. Several field studies have shown lower hygroscopicity for particles in the accumulation mode in summer, which has been attributed to a large fraction of organic mass, likely of biogenic origin^11, 13^.

The objective of our research is to elucidate the impact of seasonal changes in the types and relative amounts of BSOA and PBAPs on the cloud forming potential of aerosols in forest environments. Submicron aerosol samples were collected in the Tomakomai Experimental Forest (TOEF) in northern Japan, which is a cool-temperate mixed forest. In this study, we show the impact of seasonal changes in the WSOM composition on the cloud forming potential of submicron aerosols by using molecular tracers with positive matrix factorization (PMF) analysis^18^. We also present evidence for modification of the aerosol hygroscopicity by specific types of biogenic emissions and discuss their implications for climate.

## Results and Discussion

### Seasonal variations in aerosol hygroscopicity

**Figure 1:**
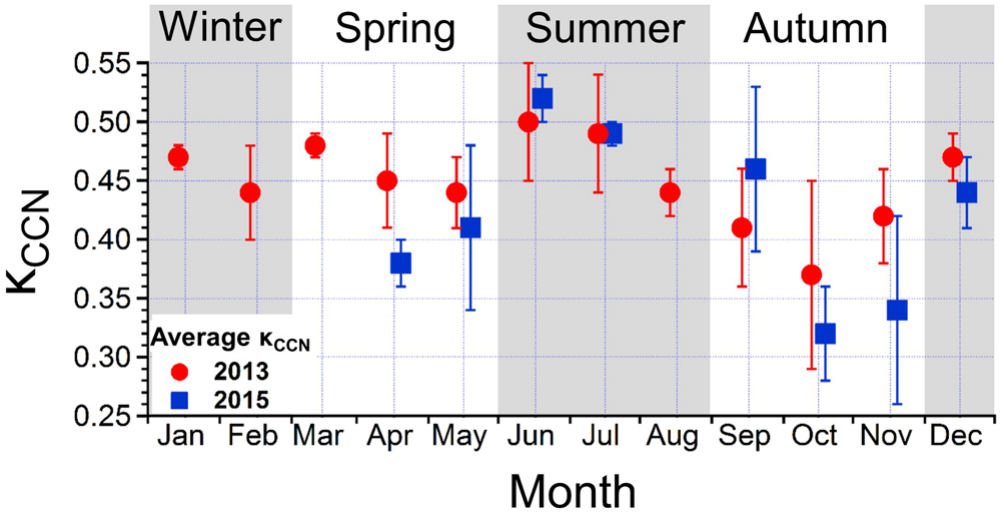
Time series of κ_CCN_ of the submicron water-soluble aerosol for the year 2013 (red circles) and 2015 (blue squares). Each season is categorized by shaded areas.

Figure 1 shows the seasonal variations in the monthly-averaged κ_CCN_ values of the submicron water-soluble aerosols in 2013 and 2015. The κ_CCN_ values ranged between 0.26 and 0.56 with an overall average of 0.44 ± 0.07. These κ_CCN_ values are generally larger than those for ambient particles obtained by *in-situ* measurements in other forest environments (typically below 0.3)^8–16^. This is expected, given that the aerosol from filter extracts is completely water-soluble, as well as because the observed aerosols at this study site were not purely organics, as discussed below. The κ_CCN_ values exhibited maxima in summer (June–August) and minima in autumn (September–November). This seasonal trend in κ_CCN_ was observed in both 2013 and 2015. The κ_CCN_ values in summer (0.50–0.52) are close to typical values of hygroscopic sulfate aerosols (~0.6). In contrast, the lower values observed in autumn (0.32–0.37) are similar to those for particles dominated by organics in forest environments, which typically range from 0.1 to 0.4^8–16^.

While the seasonal pattern of κ is highly dependent on the location, Sihto *et al*.^12^ reported the lowest hygroscopicity in boreal forest in November, which they explained to be the result of a change in the chemical composition of aerosols. Paramonov *et al*.^11^ showed a seasonal variation in κ_CCN_ with a minimum (~0.2) in July and a maximum (0.74) in February with *in-situ* ambient measurements during a 29-month period in a boreal forest in southern Finland. Levin *et al*.^13^ also observed minimum κ_CCN_ values of 0.16 in July and September, and a maximum of 0.3 in April. Those previous studies suggested that the low hygroscopicity in summer was due to a larger organic mass fraction, which is likely attributed to increased emissions of BVOCs and subsequent formation of SOA. However, they have limited chemical measurements available to confirm this, although they mentioned that a change in the chemical composition of aerosols potentially affects κ. Because κ_CCN_ is closely linked to the chemical characteristics of aerosol^4^, our observations together with the previous studies in the forested areas suggest that a seasonal difference in the chemical composition could control the seasonal variation in κ_CCN_.

### Seasonal trends of water-soluble organics and sulfate in submicron aerosols

**Figure 2:**
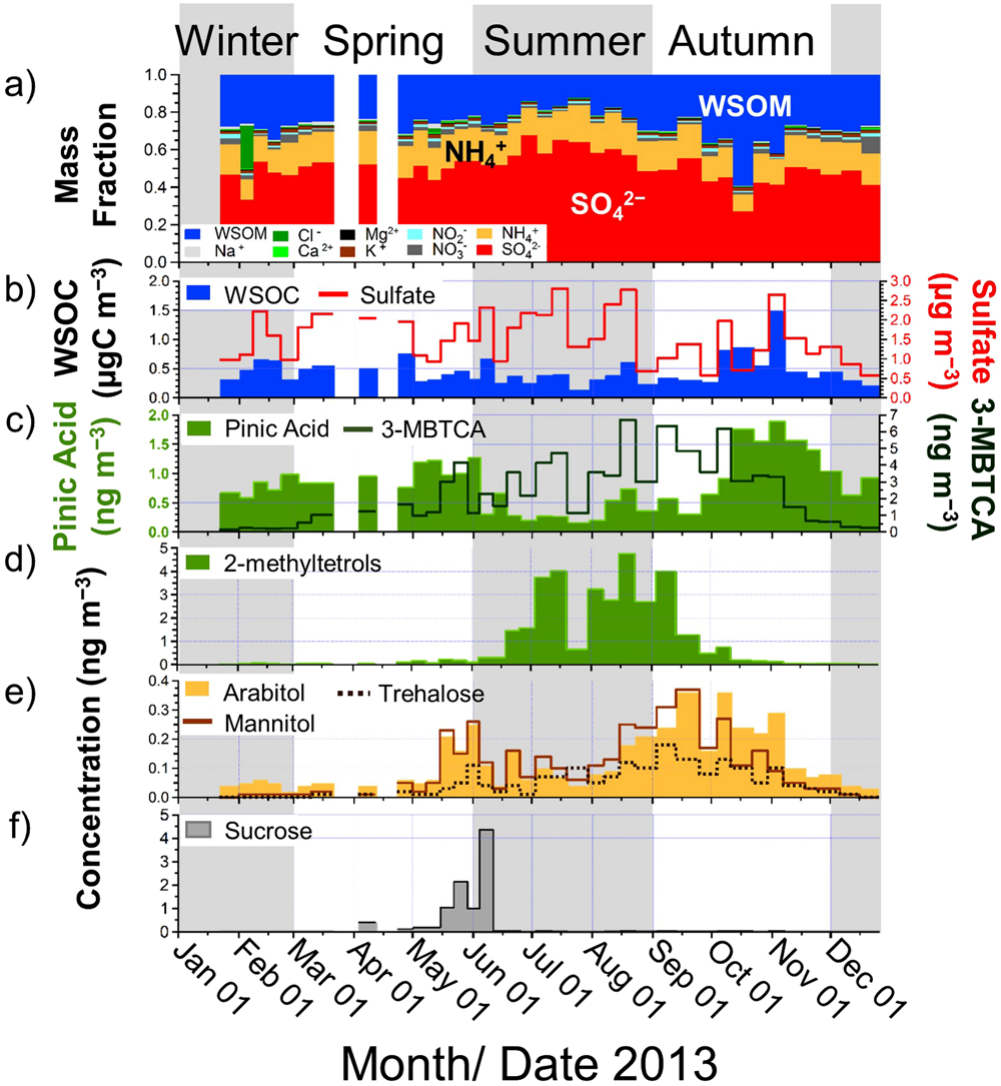
Time series of (a) the mass fraction of the chemical components in water-soluble aerosols, the mass concentrations of (b) WSOC and sulfate, (c) pinic acid and 3-methyl-1,2,3-butanetricarboxylic acid (3-MBTCA), (d) 2-methyltetrols, (e) arabitol, trehalose, and mannitol, and (f) sucrose for the year 2013. Each season is categorized by shaded areas.

**Figure 3:**
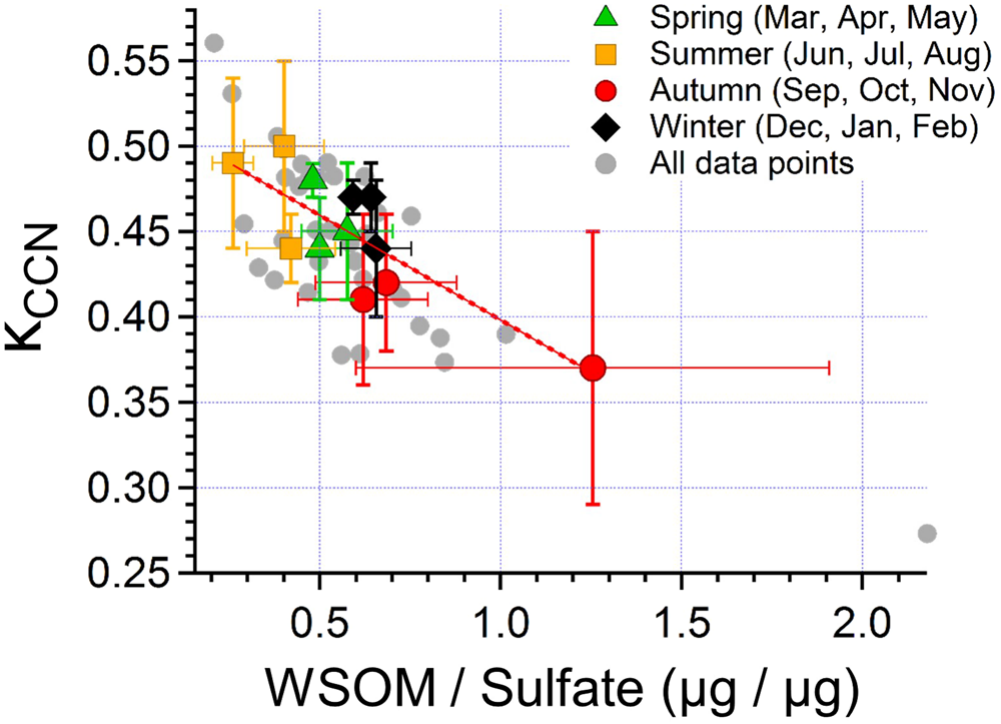
Scatter plot of monthly average κ_CCN_ and the WSOM-to-sulfate ratios with their standard deviations in spring (green triangles), summer (yellow squares), autumn (red circles), and winter (black diamonds) for the year 2013. Individual data are also shown in grey solid circles obtained for each sampling duration of approximately one week.

In order to investigate the chemical composition of the observed submicron particles, Fig. 2a–b present seasonal changes in the mass fractions of water-extracted aerosols together with the mass concentrations of water-soluble organic carbon (WSOC) and sulfate in 2013. On average, sulfate was the dominant component in summer, accounting for ~59% of the submicron water-soluble aerosols. In contrast, the mass fractions (~35%) and concentrations of WSOM (WSOC) showed maxima in autumn (Table 1). These seasonal patterns were also observed in the year 2015 (Supplementary material, Table S1). Figure 3 displays the relationship between κ_CCN_ and the WSOM-to-sulfate ratios. Throughout the entire period of 2013, the κ_CCN_ value is negatively correlated with the WSOM/sulfate ratios (R^2^ = 0.60 and 0.66 for all the individual data and monthly average data, respectively). The R^2^ values were also as large as 0.79 (all data) and 0.76 (monthly averages) for the data obtained in 2015. This suggests that the fraction of organics relative to sulfate is an important factor that controls the CCN activity of the submicron water-soluble aerosols at the study site.

**Table 1:** Average κ_CCN_ and mass concentrations of WSOM, inorganic species, and biogenic molecular tracers, together with the ambient temperature in each season for the year 2013. Individual seasonal category is defined as each three months period: Spring (March–May), Summer (June–August), Autumn (September–November), and Winter (December–February).

	Spring	Summer	Autumn	Winter
κ_CCN_	0.46 ± 0.03	0.47 ± 0.05	0.40 ± 0.06	0.45 ± 0.03
WSOM (µg m^−3^)	0.82 ± 0.26	0.66 ± 0.27	1.07 ± 0.64	0.76 ± 0.30
NO_3_^−^ (µg m^−3^)	0.07 ± 0.03	0.03 ± 0.01	0.05 ± 0.02	0.07 ± 0.03
SO_4_^2−^ (µg m^−3^)	1.65 ± 0.41	1.89 ± 0.68	1.35 ± 0.57	1.19 ± 0.51
Na^+^ (µg m^−3^)	0.03 ± 0.02	0.01 ± 0.00	0.02 ± 0.00	0.02 ± 0.01
NH_4_^+^ (µg m^−3^)	0.57 ± 0.12	0.56 ± 0.20	0.45 ± 0.19	0.36 ± 0.10
K^+^ (µg m^−3^)	0.04 ± 0.01	0.02 ± 0.01	0.04 ± 0.02	0.04 ± 0.01
2-methyltetrols (ng m^−3^)	0.12 ± 0.06	2.32 ± 1.49	0.72 ± 1.16	0.04 ± 0.02
Pinic acid (ng m^−3^)	1.01 ± 0.17	0.36 ± 0.19	1.16 ± 0.52	0.77 ± 0.14
3-MBTCA (ng m^−3^)	1.66 ± 1.09	3.29 ± 1.50	3.34 ± 1.94	0.23 ± 0.05
Sucrose (ng m^−3^)	0.57 ± 0.67	0.42 ± 1.25	0.02±0.01	0.00 ± 0.00
Trehalose (ng m^−3^)	0.03 ± 0.03	0.06 ± 0.03	0.09 ± 0.05	0.00 ± 0.00
Arabitol (ng m^−3^)	0.10 ± 0.08	0.10 ± 0.05	0.21 ± 0.10	0.04 ± 0.01
Mannitol (ng m^−3^)	0.09 ± 0.09	0.13 ± 0.07	0.16 ± 0.11	0.01 ± 0.00
Temperature (°C)	4.5 ± 3.8	18.2 ± 3.2	12.2 ± 5.4	−3.1 ± 3.2

It should be noted that even during periods with similar WSOM-to-sulfate mass ratios of 0.62–0.64, κ_CCN_ differed between autumn (0.41–0.42) and winter (0.44–0.47) (Fig. 3), which indicates that the difference in the chemical composition of WSOM can modify κ_CCN._ Specifically, it is suggested that WSOM in autumn contains less hygroscopic compounds than during the winter period. To explore the possible sources of WSOM and the resulting chemical compositions, Fig. 2c–f present the seasonal variations in the concentrations of representative tracers for BSOA and PBAPs. It is apparent that the concentrations of pinic acid, which is a first-generation oxidation product of α-pinene and is typically used as a tracer for α-pinene derived SOA^19, 20^, reach a maximum in autumn (Fig. 2c). Moreover, the concentrations of arabitol, trehalose, and mannitol also exhibited maxima during the same season (Fig. 2e), although the appearance of their peaks is slightly different from that of pinic acid. These sugar compounds are used as tracers of biological materials, including fungi and bacteria^21, 22^. In particular, trehalose is known to be a fungal metabolite as well as a stress protectant for the soil microbial community, and has been proposed as a molecular marker for fugitive dust from biologically active surface soils^23^. The seasonal profiles indicate that the increase of WSOM during the autumn period can be associated with increased concentrations of both α-pinene derived SOA and PBAPs.

Previous studies have suggested that α-pinene has been found to originate from roots, litter, and soil microbial activity^24–26^ in combination with high nitrification rates^27^. Local wind speeds were relatively low average 3.2 ± 0.5 m s^−1^ with a prevailing northerly wind (Supplementary material, Fig. S1) from the forested area in autumn. This supports the hypothesis of a significant influence of local emissions and subsequent vertical transport of α-pinene and its oxidation products within the canopy. Moreover, the enhanced concentrations of pinic acid together with these sugar compounds in autumn indicate that most of the observed pinic acid, and thus its precursor (α-pinene), in this season originated from the forest floor rather than from the leaves.

The concentrations of 2-methyltetrols, which is used as a tracer for isoprene-derived SOA^28^, showed a maximum in summer when the sulfate fraction dominated as described above. The seasonal trend of 2-methyltetrols (Fig. 2d) closely followed that of the ambient temperature (Table 1). The increased sulfate concentration is likely due to the higher influence of anthropogenic emissions originating from the industrial area of the Tomakomai city located in the south of the forest site. This is supported by the predominant southerly wind direction in summer (Supplementary material, Fig. S1).

### Source apportionment of WSOC by positive matrix factorization (PMF) analysis

We performed a PMF analysis to apportion sources of the measured WSOC, which is closely linked to κ_CCN_. The PMF resolved five interpretable factors, which were characterized by the enrichment of each tracer compound (Supplementary material, Fig. S2). Factor 1 (F1) was dominated by the contribution of sucrose (89%), which is a primary saccharide of pollen grains. Consequently, it is referred to here as “pollen-rich”. Factor 2 (F2) is characterized by 2-methyltetrols (86%), whereas Factor 3 (F3) was dominated by pinic acid (75%). On the basis of the characteristics of each source profile, F2 and F3 are referred to here as “isoprene-SOA-rich” and “less oxidized α-pinene-SOA-rich”, respectively. Factor 4 (F4) is characterized by large contributions of mannitol (75%), arabitol (74%), trehalose (60%), and 3-methyl-1,2,3-butanetricarboxylic acid (3-MBTCA) (61%). It has been recognized that 3-MBTCA is a highly oxidized compound of α-pinene^29^. Although F4 was difficult to convincingly attribute to a specific source, given the possibility that these tracers for PBAP are associated with re-suspended soil and associated biota and that 3-MBTCA is a highly oxidized product of α-pinene, F4 was labeled here as “mixtures of fungi, bacteria, and more oxidized α-pinene SOA”. On the other hand, Factor 5 (F5) is dominated by sulfate (57%), sodium (54%), and ammonium (51%). The observed sulfate is suggested to originate from anthropogenic activity rather than from sea salt, because of the negligible fraction of sea-salt sulfate (<2%) calculated from the concentrations of sodium (Table 1). Consequently, F5 is referred to here as “anthropogenic sulfate-rich” as a possible source category of WSOC.

**Figure 4:**
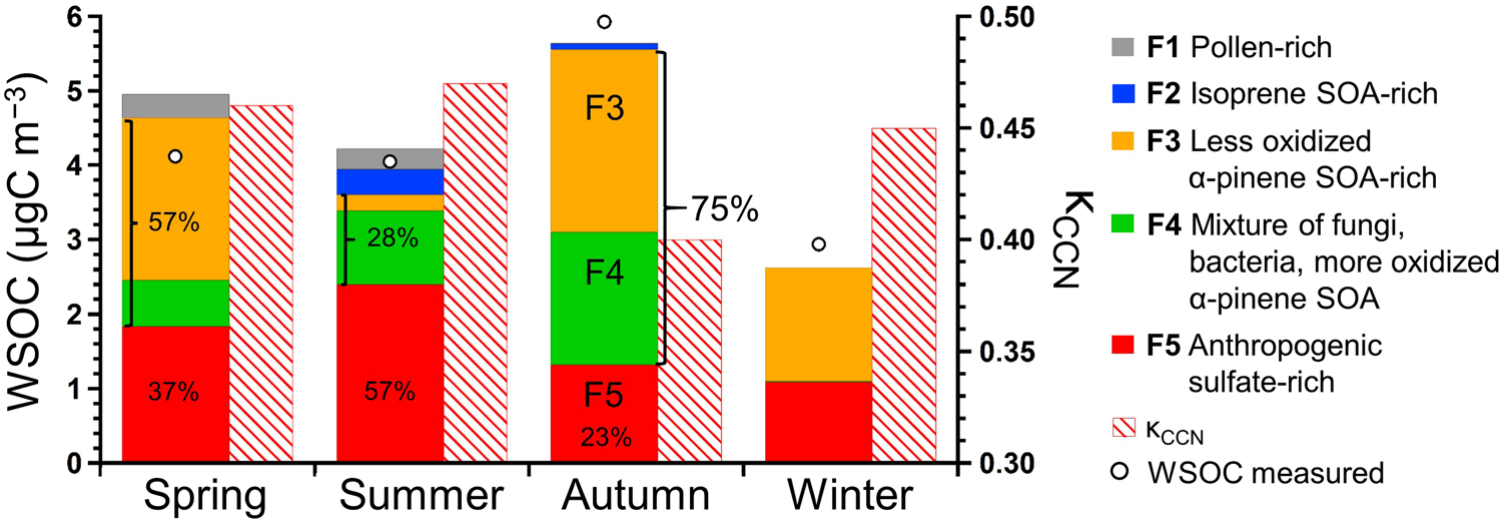
Contribution of each PMF-derived factor to the WSOC mass concentration and the average κ_CCN_ in each season. Open circles indicate the average mass concentrations of the measured WSOC.

Figure 4 displays seasonal differences in the contribution of each source factor to the WSOC mass resolved by the PMF. The results show that 98% of the measured WSOC mass concentration in 2013 was successfully reproduced by the PMF model. In autumn, 75% of the WSOC mass was attributable to the sum of F3 and F4, both of which are dominated by the contribution of α-pinene derived SOA and fungi/bacteria derived WSOC. The significant contribution of α-pinene derived SOA was also apparent in spring, when the sum of F3 and F4 accounted for 57% of WSOC mass on average. The pollen-rich factor (F1) contributed only a minor fraction of the submicron WSOC, even in spring and summer (<7%). In summer, the anthropogenic sulfate-rich factor (F5) contributed significantly to WSOC mass (~57%), whereas the contribution of α-pinene derived SOA and fungi/bacteria derived organics to the WSOC was smaller (~28%). The increased concentrations of sulfate can also facilitate the formation of highly oxidized, hydrophilic OA, which implies that the formation of highly oxidized organics could have been a major component of observed WSOC in summer. In contrast, the WSOC in winter was attributed to less oxidized α-pinene SOA and anthropogenic sulfate-rich factors, with the lowest average concentration through the year. The present result indicates that the relative abundance and contributions of different sources to the WSOC mass are important for controlling the variation of the CCN activity.

### Factors controlling the hygroscopicity parameter at the forest site

The modification of κ_CCN_ associated with the increased contributions of α-pinene SOA is supported by previous studies. Oxidation products of α-pinene are known to be surfactants and as such, when mixed with inorganic salts, they have been found to affect the surface tension of the solution^30–32^. In general, depression in the surface tension caused by surfactants lowers the Kelvin (surface tension) effects^33^, which leads to increasing CCN activity^31, 33, 34^. On the other hand, surfactants also affect the Raoult (water activity) effects, which can compensate the Kelvin effects^30, 31, 35^. In mixed particles, κ can be modelled as the volume weighted average of the constituent’ κ values, which is estimated based on volume-mixing rules^4^. The estimated κ (κ_mix_) is defined as κ_mix_ = ∑ɛ_*i*_κ_*i*_, where ɛ_*i*_ and κ_i_ are the volume fraction and the κ value of each constituent, respectively. When κ_mix_ is simply determined for two-component mixtures of WSOM and ammonium sulfate (Table 1 and also see the method section), the observed κ_CCN_ and predicted κ_mix_ values agreed to within 17%, which is consistent with previous studies^8, 10, 32, 36^. It is noted that the κ_CCN_ shown here is the apparent κ, which is sensitive to the assumption used in the calculations (equations (1) and (2)) and is different from intrinsic κ^37^. Padró *et al*.^32^ evaluated κ for water-extracted aerosols in Mexico City by assuming the surface tension of water and 15% suface tension from the contributions of organics. They demonstrated that when κ_CCN_ is calculated allowing for a 15% surface tension depression, surfactant characteristics of the water-soluble aerosol can result in a reduction in organic hygroscopicity.

The reduction in hygroscopicity can be explained with an increased fraction of surface active organic compounds^30, 31, 35^_._ While surfactants generally reduce the surface tension effect (Kelvin effect) of the droplet, which leads to increased CCN activity^33, 34^, subsequent phase partitioning and/or organic coating lead to an organic-rich phase on the surface of the droplet and inner aqueous phase. This process can compensate for the surface tension effect if the fraction of surfactants is large. The surfactants lower the Raoult (water activity) effects^31, 35^, which may result in a reduction in CCN activity^30, 31, 35^. In recent laboratory experiments, Renbaum-Wolff *et al*.^38^ demonstrated that internally mixed sulfate and SOA, enriched with the oxidation products of α-pinene, results in a particle with a liquid-liquid phase separation. Furthermore, they showed that phase partitioning occurs with a phase enriched with α-pinene secondary OM on the surface of the particle and the water-rich inner phase above 95% RH. This surface organic-rich phase can prevent particles from activating as CCN. This mechanism can partly explain the reduction of κ_CCN_ in autumn, when the mass fraction of WSOM, dominated by α-pinene derived SOA, was large relative to that of sulfate.

It is interesting to note that F3 has a significant contribution of nitrate (53%; Supplementary material, Fig. S2). In fact, the concentration of nitrate showed a positive correlation with that of pinic acid (R^2^ = 0.26) in autumn, whereas there was an insignificant correlation with benzoic acid (R^2^ = 0.04; data not shown in this paper), which is measured as a tracer of anthropogenic origin. These results, together with low local wind speeds and prevailing wind direction from the forest area (Supplementary material, Fig. S1), indicate that a substantial fraction of the NO_x_ originated from soil^27^ in the forest rather than from anthropogenic sources, to form aerosol nitrate. Nitrate radicals in the atmosphere are known to have a high reactivity to α-pinene and can subsequently form α-pinene nitrates^39^. Recently, chamber experimental and modelling studies have shown that the addition of nitrate functional groups inhibit CCN activation by reducing its miscibility with water^40, 41^, which also supports the observed reduction in CCN activity for particles in the vicinity of a biogenic source area.

In summary, our measurements indicate that in cool-temperate forests, where isoprene emissions are relatively low, the formation of α-pinene-derived SOA associated with soil/litter mostly found near the forest floor, can significantly contribute to reducing the cloud forming potential of submicron particles. Additional field studies are needed to investigate the mechanism for organics from the forest floor reducing the CCN activity. Future work should include more sample sets from multiple forest sites and provide comprehensive *in-situ* measurements of size distribution, chemical compositions, and CCN concentrations to evaluate the relationships of these physicochemical characteristics. The present findings might have important implications, and demonstrate that the seasonal variation in κ controlled by different types of biogenic sources is significant and should be taken into account in similar forest environments. They are also important for predicting regional climate effects due to changes in types and amounts of biogenic emissions particularly in cool-temperate/boreal forests in the future.

## Methods

### Location and submicron aerosol sampling

Ambient submicron aerosol samples were collected in Hokkaido University’s 2715 ha Tomakomai Experimental Forest (TOEF) (42°43′N, 141°36′E) located in the southwestern part of Hokkaido, northern Japan in the cool-temperate zone. The southern boundary of the forest site faces Tomakomai city and its industrial port area towards the Pacific Ocean. The mixed cool temperate forest consists of mature and secondary deciduous forest, and manmade coniferous forest with various types of forest floor covers. Tree species include Mongolian oak (*Quercus crispula*), mono maple (*Acer mono*), Korean mountain ash (*Sorbus alnifolia*), Japanese linden (*Tilia japonica*) and the planted species Japanese larch (*Larix leptolepsis*), sakhalin fir (*Abies sachalinensis*), and sakhalin spruce (*Picea glehnii*)^42^. The soil consists of volcanogenic regosols, which are shallow and less weathered^43^. The meteorological data for the sampling site was obtained from the Japan Meteorological Agency (available at http://www.jma.go.jp/jma/index.html). The predominant local wind direction in autumn and winter was from the north, corresponding to the forested area (Supplementary material, Fig. S1). In contrast, fractions of air transported from the south (coastal urban area) were dominant in summer. The monthly averaged temperature ranged from −1.9 ± 2.9 °C (winter) to 18.2 ± 3.1 °C (summer) for the years 2013 and 2015.

Submicron aerosol samples were collected continuously using a high-volume air sampler (HVAS; Model 120SL, Kimoto Electric, Osaka, Japan) at an altitude of ~18 m above the forest floor at the research site. A cascade impactor (CI; Model TE-234, Tisch Environmental, Cleves, OH, USA) attached to the HVAS was used to collect size-segregated particles^44^ with a flow rate of 1130 L min^−1^. In this study, we only used analytical results obtained from the bottom stage of the impactor, which collected particles with aerodynamic diameter smaller than 0.95 μm. The sampling duration of each aerosol sample was approximately 1 week. The samples were collected on quartz fiber filters (25 cm  × 20 cm) from January to December in 2013, and from February to December in 2015. Quartz fiber filters were pre-combusted at 410 °C for 6 hours to remove any contaminants. Collected filters were individually stored in glass jars with a Teflon-lined screwed cap at −20 °C to limit chemical reactions on the filter and losses of volatile compounds. In total, 37 samples for 2013 and 15 samples for 2015 have been analyzed, with the focus on 2013 presented in this study.

### Hygroscopicity measurement and determination of κ_CCN_

To measure the hygroscopic parameters of submicron water-soluble aerosols, a filter cut of 0.79 cm^2^ was extracted with 7 mL ultrapure water using an ultrasonic bath (5 min × 3 times). The extracts were filtered through a 0.22 µm pore syringe filter (Millex-GV, 0.22 μm, Millipore) to remove any insoluble particles with diameters larger than 0.22 µm. Polydisperse aerosols were generated by first atomizing the filter extracts and then drying them with two diffusion dryers (silica gel and molecular sieve) in series. After passing the impactor and bipolar charger of the electrostatic classifier (TSI Model 3080), particles with a specific dry mobility diameter (D_dry_) were selected by a differential mobility analyzer (DMA, TSI Model 3081). The classified flow was then split into two parallel streams: one went into the condensation particle counter (CPC, TSI Model 3775) to measure the total concentration of condensation nuclei (N_CN_), whereas the other stream was channeled into a continuous-flow thermal-gradient diffusion chamber (CCN-100, Droplet Measurement Technologies) to measure the number concentration of CCN (N_CCN_)^45^. The flow rate into the CCN counter was 0.5 L min^−1^ with a sheath-to-sample flow ratio of 10.

In order to measure size-resolved CCN activity and growth kinetics, Scanning Mobility CCN Analysis (SMCA) was performed^46^. In short, the method involves scanning a dynamic mobility diameter ranging from 10.4 to 220.7 nm with the DMA over a time period of 255 s. The specific supersaturation (SS, 0.25% to 1.0%) in the CCN counter was kept constant during the scan. The CCN activity of the particles generated was characterized by the activation diameter (D_act_) at the corresponding SS, where the D_act_ of CCN is defined as the D_dry_ at which N_CCN_ reaches 50% of the total N_CN_ at a certain SS^47^. D_act_ can be determined by expressing the ratio of N_CCN_ to N_CN_ with respect to D_dry_ and fitting the data to a sigmoid curve^47^. A multiple-charge correction was applied to the N_CCN_ and N_CN_ using the algorithm provided in Moore *et al*.^46^. The algorithm re-bins misclassified multiply charged particles into their actual size bins based on an equilibrium charge distribution.

The hygroscopicity parameter, κ_CCN_, can be calculated for particles with D_dry_ at a certain SS as follows^4^:

1 $${\rm{\kappa }}(\mathrm{SS},{{\rm{D}}}_{{\rm{dry}}})\approx \frac{4{{\rm{A}}}^{3}}{27{{\rm{D}}}_{{\rm{dry}}}^{3}{\mathrm{ln}}^{2}{{\rm{SS}}}_{{\rm{C}}}}$$

2 $${\rm{A}}=\frac{4{\sigma }_{{\rm{s}}/{\rm{a}}}{{\rm{M}}}_{{\rm{w}}}}{{\rm{R}}{\rm{T}}{\rho }_{{\rm{w}}}}$$

where SS_C_ represents the critical supersaturation for activation, ρ_w_ is the density of water, M_w_ is the molar mass of water, σ_s/a_ is the surface tension of the pure water–air interface (σ_s/a_ = 0.072 J m^−2^), R is the universal gas constant, and T is the absolute temperature (T = 298 K). Calibration was performed with ammonium sulfate before and after the measurements. The hygroscopicity data are presented in the Supplementary material (Tables S3 and S4).

### Chemical analysis of water-soluble aerosols

The term water-soluble aerosols in the present study is technically defined as particles sampled on the filter and extracted with ultrapure water followed by being filtered through the syringe filter^44, 48^. To determine the WSOC concentration of the PM_1.0_ filter samples, another filter cut of 3.14 cm^2^ was extracted with 20 mL ultrapure water using an ultrasonic bath for 15 min. The extracts were filtered through the same type of 0.22 µm pore syringe filter as described above, before being injected into a total organic carbon analyzer (Model TOC-L_CHP_, Shimadzu). The mass concentrations of WSOC were converted to those of water-soluble organic matter (WSOM) using a conversion factor of 1.8^49, 50^.

Another portion of the filter (3.80 cm^2^) was extracted with dichloromethane/methanol to measure biogenic molecular tracers: 2-methyltetrols (the sum of 2-methylerythritol and 2-methylthreitol)^28^, pinic acid, 3-methyl-1,2,3-butanetricarboxylic acid (3-MBTCA)^19, 20, 29^, trehalose, arabitol, mannitol, and sucrose^21, 22^. The –COOH and –OH functional groups in the extracts were reacted with N,O-bis-(trimethylsilyl) trifluoroacetamide (BSTFA) to form trimethylsilyl (TMS) esters and TMS ethers, respectively. The TMS derivatives were then analyzed for the compounds listed above using a capillary gas chromatograph (GC7890, Agilent) coupled to a mass spectrometer (MSD5975C, Agilent). Additionally, another cut of the filter (3.14 cm^2^) was extracted with 10 mL of ultrapure water under ultrasonication to determine the concentration of major inorganic ions (NO_3_^−^, SO_4_^2−^, Na^+^, NH_4_^+^, K^+^). The same syringe filter type as described above was used, before the extract was injected into the ion chromatograph (Model 761 compact IC; Metrohm)^51^. The data for the chemical measurements are given in the Supplementary material (Tables S5 to S7).

### Positive matrix factorization (PMF)

Positive matrix factorization (PMF)^18^ is a multivariate factor analysis technique used to resolve the identities and contributions of unknown mixtures to the observed components. Observed species are expressed as the sum of contributions from a number of time-invariant source profiles (factors) and residuals that cannot be modeled. By minimizing the residual term, the solution that best reproduces the observations can be determined. Further details of the PMF model and its application can be found elsewhere^18, 52^.

Thirteen chemical components in thirty-seven sample sets were used as input data in the PMF model for the year 2013 (Supplementary material, Fig. S2). The analytical measurement uncertainties of each component were taken into account for the calculation. The calculation was performed with 100 runs. To maximize the amount of data, missing values were replaced with the median values of the species^53^. The data from species with a signal-to-noise (S/N) ratio equal to or less than 0.5 were excluded from the analysis. Moreover, the data of species with an S/N ratio equal to or less than 1.0 were labeled as “weak”. Based on the scaled residual analysis with ±3 standard deviations, NO_3_^−^ and NH_4_^+^ were additionally labeled as “weak”. Uncertainties for these weak species are tripled^54^. To determine the best solution, three to seven factor solutions were calculated, which resulted in the selection of five factors as the best solutions.

The uncertainty of the PMF solution is estimated by displacement (DISP) analysis and bootstrapping (BS) analysis^55^. With DISP, each fitted element of the factor profile in the solution is “displaced” by predetermined error levels to determine changes, or swaps, in the factor profiles. No swaps occurred at the lowest level. With BS, multiple PMF solutions are generated by using a series of data sets that are resampled versions of the original data set. By comparing the factor contributions, each BS factor is mapped to a base factor of the original PMF solution. The threshold of the minimum correlation for mapping was 0.6, and 100 bootstrapping runs were carried out. All BS factors could be mapped, and only BS Factor 4 showed mapping to the base Factor below 100% (94%) (Supplementary material, Table S2). In this case, four of the BS runs were mapped to F1 instead of F4, likely due to the fact that both factors contain contributions of PBAP tracers.

### Estimation of the predicted κ values

The predicted hygroscopicity parameter (κ_mix_) was calculated by applying a mixing rule^4^ to the aerosol chemical composition as follows:

3 $${\kappa }_{mix}={\varepsilon }_{AS}{\kappa }_{AS}+{\varepsilon }_{org}{\kappa }_{org}$$

where κ_AS_ and κ_org_ are typical κ values for ammonium sulfate (~0.6)^4, 8^ and biogenic secondary organic aerosols (~0.1)^8, 56^, respectively. The ε_AS_ and ε_org_ values are the volume fractions of ammonium sulfate and organics, respectively, which were calculated from the mass concentrations using the density of ammonium sulfate (ρ_AS_ = 1.77 g cm^−3^) and the assumed density of organics (ρ_org_ = 1.4 g cm^−3^)^10^.

## Electronic supplementary material

Supplementary Information
