# Supplementary material for: Evidence of a reduction in cloud condensation nuclei activity of water-soluble aerosols caused by biogenic emissions in a cool-temperate forest
Source: Sci Rep. 2017 Aug 16;7:8452. doi: 10.1038/s41598-017-08112-9 (PMC5559486; doi:10.1038/s41598-017-08112-9)
Supplement: Supplementary file 1 — Supplementary Information [file 41598_2017_8112_MOESM1_ESM.pdf]

Supplementary Information for the manuscript:

Evidence of a reduction in cloud condensation nuclei activity of water-soluble aerosols caused by biogenic emissions in a cool-temperate forest

Astrid Müller[1,2], Yuzo Miyazaki[1], Eri Tachibana[1], Kimitaka Kawamura[1,3], & Tsutom Hiura[4]

1 Institute of Low Temperature Science, Hokkaido University, Sapporo, 060-0819, Japan

2 Graduate School of Environmental Science, Hokkaido University, Sapporo, 060-0810, Japan

3 Chubu Institute for Advanced Studies, Chubu University, Kasugai, 487-8501 Japan

4 Field Science Center for Northern Biosphere, Hokkaido University, Tomakomai, 053-0035, Japan

Correspondence and requests for materials should be addressed to Y. M. (email: yuzom@lowtem.hokudai.ac.jp)

Table S1. Average κ_{CCN} and mass concentrations of WSOM and inorganic species, together with the ambient temperature in each season in 2015. Individual seasonal category is defined as each three months period: Spring (March–May), Summer (June–August), Autumn (September–November), and Winter (December–February).

	Spring	Summer	Autumn	Winter
κ_{CCN}	0.40±0.06	0.51±0.02	0.37±0.09	0.44±0.03
WSOM (μg m^{-3})	1.60±0.23	0.83±0.48	2.21±2.27	0.72±0.00
NO_3^- (μg m^{-3})	0.09±0.04	0.01±0.01	0.06±0.10	0.01±0.00
SO_4^{2-} (μg m^{-3})	1.69±0.44	1.87±1.16	0.98±0.47	1.01±0.00
Na^+ (μg m^{-3})	0.06±0.01	0.01±0.01	0.01±0.01	0.01±0.00
NH_4^+ (μg m^{-3})	0.52±0.19	0.59±0.35	0.32±0.17	0.32±0.00
K^+ (μg m^{-3})	0.02±0.01	0.00±0.00	0.06±0.09	0.00±0.00
Temperature (°C)	6.6±3.8	18.0±3.0	10.9±5.6	−0.8±2.0

Table S2. Results of the analysis for the uncertainty in the five-factor solution in the PMF calculation. Q is a goodness-of-fit parameter, Q_{robust} indicates Q excluding points which did not fit. $Q_{expected}$ is the calculated Q, whereas %dQ indicates percent change in Q when swaps occur.

Diagnostic	5 Factors
$Q_{robust}/Q_{expected}$	4.56
DISP %dQ:	<0.05%
Number of DISP swaps	0
Factors with BS (100 runs) < 100%	Factor 4 (96%)
BS unmapped	0

Table S3. The average (Avg.) κ_{CCN} values and the standard deviation (Std.) for the submicron water-soluble aerosols in each sampling period for the year 2013.

		Year 2013	
**Sampling start date (MM/DD)**	**Sampling end date (MM/DD)**	**Avg. κ_{CCN}**	**Std.**
01/22	02/01	0.47	0.01
02/01	02/08	0.39	0.01
02/08	02/15	0.48	0.01
02/15	02/22	0.41	0.03
02/22	03/03	0.44	0.02
03/03	03/10	0.48	0.01
03/10	03/21	0.48	0.02
04/03	04/12	0.49	0.01
04/23	05/01	0.42	0.01
05/01	05/08	0.41	0.01
05/08	05/15	0.43	0.02
05/15	05/22	0.45	0.01
05/22	05/29	0.48	0.01
05/29	06/04	0.50	0.01
06/04	06/11	0.49	0.02
06/11	06/18	0.43	0.02
06/18	06/25	0.51	0.02
06/25	07/03	0.56	0.02
07/03	07/11	0.43	0.01
07/11	07/19	0.53	0.02
07/19	07/30	0.51	0.02
07/30	08/07	0.42	0.01
08/07	08/15	0.45	0.01
08/15	08/23	0.44	0.01
08/23	09/03	0.42	0.02
09/03	09/13	0.38	0.02
09/13	09/25	0.48	0.02
09/25	10/04	0.37	0.02
10/04	10/11	0.46	0.01
10/11	10/22	0.27	0.00
10/22	10/30	0.39	0.01
10/30	11/07	0.39	0.01
11/07	11/18	0.45	0.01
11/18	11/25	0.38	0.01
11/25	12/06	0.45	0.01
12/06	12/16	0.48	0.01
12/16	12/26	0.46	0.01

Table S4. The average (Avg.) κ_{CCN} values and the standard deviation (Std.) for the submicron water-soluble aerosols in each sampling period for the year 2015.

		**Year 2015**	
**Sampling start date (MM/DD)**	**Sampling end date (MM/DD)**	**Avg. κ_{CCN}**	**Std.**
4/14	4/23	0.38	0.02
5/7	5/19	0.34	0.01
5/19	5/28	0.48	0.01
5/28	6/9	0.51	0.01
6/19	6/26	0.53	0.02
7/7	7/16	0.49	0.01
7/27	8/3	0.50	0.01
9/11	9/18	0.38	0.01
9/18	9/28	0.52	0.02
9/28	10/9	0.37	0.01
10/19	10/28	0.28	0.01
10/28	11/5	0.26	0.01
11/5	11/13	0.33	0.01
11/13	12/16	0.44	0.01
12/16	12/22	0.44	0.03

Table S5. The mass concentrations of biogenic molecular tracers during each sampling period for the year 2013.

Year 2013								
**Sampling start date (MM/DD)**	**Sampling end date (MM/DD)**	**2-methyl-tetrols (ng m^{-3})**	**Pinic acid (ng m^{-3})**	**3-MBTCA (ng m^{-3})**	**Sucrose (ng m^{-3})**	**Trehalose (ng m^{-3})**	**Arabitol (ng m^{-3})**	**Mannitol (ng m^{-3})**
01/22	02/01	0.03	0.67	0.15	0.00	0.00	0.04	0.00
02/01	02/08	0.04	0.58	0.24	0.00	0.00	0.05	0.01
02/08	02/15	0.08	0.85	0.22	0.00	0.00	0.06	0.01
02/15	02/22	0.05	0.71	0.18	0.01	0.00	0.05	0.01
02/22	03/03	0.03	0.98	0.22	0.00	0.00	0.02	0.01
03/03	03/10	0.04	0.83	0.56	0.00	0.00	0.04	0.01
03/10	03/21	0.06	0.84	1.01	0.01	0.01	0.05	0.02
04/03	04/12	0.07	0.96	1.26	0.41	0.01	0.04	0.01
04/23	05/01	0.12	0.77	1.66	0.13	0.02	0.06	0.05
05/01	05/08	0.18	1.19	0.97	0.17	0.01	0.05	0.02
05/08	05/15	0.07	1.22	1.18	0.20	0.01	0.06	0.05
05/15	05/22	0.23	0.98	3.01	1.05	0.03	0.21	0.23
05/22	05/29	0.19	1.00	4.15	2.15	0.05	0.16	0.15
05/29	06/04	0.10	1.27	1.13	1.00	0.11	0.25	0.26
06/04	06/11	0.31	0.30	2.29	4.37	0.04	0.11	0.12
06/11	06/18	0.30	0.66	1.56	0.04	0.02	0.04	0.03
06/18	06/25	1.46	0.28	3.55	0.04	0.04	0.16	0.16
06/25	07/03	1.58	0.20	2.15	0.01	0.01	0.06	0.07
07/03	07/11	3.76	0.27	4.12	0.05	0.07	0.10	0.14
07/11	07/19	4.02	0.27	4.73	0.02	0.07	0.07	0.10
07/19	07/30	0.65	0.16	1.14	0.00	0.10	0.04	0.06
07/30	08/07	3.24	0.20	3.59	0.00	0.05	0.08	0.11
08/07	08/15	2.77	0.54	3.37	0.02	0.07	0.09	0.13
08/15	08/23	4.76	0.73	6.72	0.03	0.12	0.18	0.25

08/23	09/03	2.70	0.36	3.00	0.02	0.10	0.21	0.24
09/03	09/13	4.02	0.57	6.34	0.03	0.18	0.24	0.31
09/13	09/25	1.28	0.30	4.85	0.02	0.13	0.36	0.37
09/25	10/04	0.48	0.64	3.59	0.02	0.08	0.16	0.17
10/04	10/11	0.75	0.91	6.19	0.03	0.13	0.36	0.27
10/11	10/22	0.20	1.75	3.03	0.03	0.10	0.24	0.11
10/22	10/30	0.17	1.55	3.38	0.01	0.05	0.22	0.16
10/30	11/07	0.15	1.89	3.29	0.05	0.10	0.29	0.09
11/07	11/18	0.06	1.57	1.47	0.01	0.04	0.10	0.05
11/18	11/25	0.04	1.40	0.69	0.01	0.03	0.07	0.03
11/25	12/06	0.05	1.04	0.59	0.01	0.02	0.08	0.03
12/06	12/16	0.04	0.63	0.32	0.01	0.01	0.04	0.01
12/16	12/26	0.02	0.93	0.27	0.01	0.00	0.03	0.00

Table S6. The mass concentrations of WSOC (WSOM) and inorganic species during each sampling period for the year 2013.

				Year 2013				
**Sampling start date (MM/DD)**	**Sampling end date (MM/DD)**	**WSOC (μgC m^{-3})**	**WSOM (μg m^{-3})**	**Nitrate (μg m^{-3})**	**Sulfate (μg m^{-3})**	**Sodium (μg m^{-3})**	**Ammonium (μg m^{-3})**	**Potassium (μg m^{-3})**
01/22	02/01	0.32	0.58	0.07	0.98	0.03	0.34	0.03
02/01	02/08	0.48	0.86	0.07	1.11	0.05	0.36	0.05
02/08	02/15	0.67	1.20	0.04	2.23	0.02	0.56	0.05
02/15	02/22	0.64	1.16	0.04	1.60	0.03	0.40	0.05
02/22	03/03	0.32	0.57	0.09	0.98	0.01	0.36	0.04
03/03	03/10	0.50	0.89	0.06	1.81	0.04	0.59	0.06
03/10	03/21	0.56	1.01	0.06	2.16	0.08	0.66	0.04
04/03	04/12	0.51	0.92	0.11	2.04	0.04	0.70	0.04
04/23	05/01	0.76	1.37	0.12	1.96	0.03	0.75	0.06
05/01	05/08	0.28	0.51	0.04	1.09	0.01	0.40	0.03
05/08	05/15	0.31	0.56	0.07	0.94	0.05	0.37	0.04
05/15	05/22	0.40	0.72	0.08	1.47	0.01	0.54	0.05
05/22	05/29	0.47	0.85	0.05	1.91	0.02	0.61	0.02
05/29	06/04	0.32	0.58	0.06	1.46	0.02	0.49	0.03
06/04	06/11	0.67	1.21	0.05	2.32	0.01	0.80	0.02
06/11	06/18	0.26	0.47	0.04	0.94	0.00	0.29	0.04
06/18	06/25	0.38	0.69	0.04	1.80	0.01	0.55	0.03
06/25	07/03	0.25	0.45	0.03	2.18	0.01	0.46	0.03
07/03	07/11	0.39	0.70	0.04	2.13	0.01	0.70	0.02
07/11	07/19	0.40	0.72	0.03	2.80	0.01	0.67	0.03
07/19	07/30	0.14	0.25	0.02	1.30	0.01	0.40	0.02
07/30	08/07	0.32	0.57	0.03	1.52	0.01	0.42	0.02
08/07	08/15	0.39	0.70	0.03	2.39	0.01	0.76	0.02
08/15	08/23	0.61	1.10	0.04	2.77	0.02	0.84	0.03
08/23	09/03	0.24	0.43	0.02	0.69	0.00	0.23	0.02

09/03	09/13	0.35	0.62	0.03	1.02	0.01	0.33	0.03
09/13	09/25	0.31	0.56	0.03	1.38	0.01	0.45	0.02
09/25	10/04	0.27	0.49	0.03	0.58	0.01	0.18	0.02
10/04	10/11	0.82	1.48	0.06	1.97	0.02	0.68	0.06
10/11	10/22	0.86	1.56	0.03	0.71	0.01	0.23	0.05
10/22	10/30	0.56	1.01	0.07	1.21	0.02	0.44	0.04
10/30	11/07	1.49	2.69	0.04	2.65	0.02	0.86	0.10
11/07	11/18	0.45	0.81	0.05	1.53	0.02	0.52	0.03
11/18	11/25	0.35	0.64	0.04	1.14	0.02	0.37	0.04
11/25	12/06	0.46	0.82	0.08	1.30	0.02	0.48	0.04
12/06	12/16	0.30	0.54	0.03	0.87	0.01	0.27	0.03
12/16	12/26	0.21	0.38	0.13	0.58	0.01	0.24	0.02

Table S7. The mass concentrations of WSOC (WSOM) and inorganic species during each sampling period for the year 2015.

Sampling start date (MM/DD)	Sampling end date (MM/DD)	WSOC (μgC m^{-3})	WSOM (μg m^{-3})	Year 2015<br>Nitrate (μg m^{-3})	Sulfate (μg m^{-3})	Sodium (μg m^{-3})	Ammonium (μg m^{-3})	Potassium (μg m^{-3})
4/14	4/23	1.06	1.90	0.11	1.28	0.05	0.49	0.04
5/7	5/19	0.85	1.53	0.11	1.50	0.07	0.31	0.02
5/19	5/28	0.76	1.36	0.04	2.30	0.06	0.77	0.00
5/28	6/9	0.07	0.13	0.00	0.13	0.00	0.04	0.00
6/19	6/26	0.37	0.66	0.01	2.01	0.00	0.64	0.00
7/7	7/16	0.65	1.17	0.03	1.96	0.02	0.67	0.00
7/27	8/3	0.76	1.37	0.01	3.40	0.00	1.02	0.00
9/11	9/18	0.47	0.85	0.01	0.66	0.02	0.19	0.02
9/18	9/28	0.41	0.74	0.01	1.67	0.01	0.46	0.00
9/28	10/9	0.38	0.69	0.01	0.43	0.02	0.11	0.00
10/19	10/28	1.60	2.88	0.05	0.82	0.01	0.25	0.10
10/28	11/5	4.16	7.50	0.30	1.66	0.01	0.64	0.26
11/5	11/13	0.94	1.69	0.04	0.57	0.01	0.19	0.03
11/13	12/16	0.62	1.12	0.03	1.08	0.01	0.38	0.00
12/16	12/22	0.40	0.72	0.01	1.01	0.01	0.32	0.00

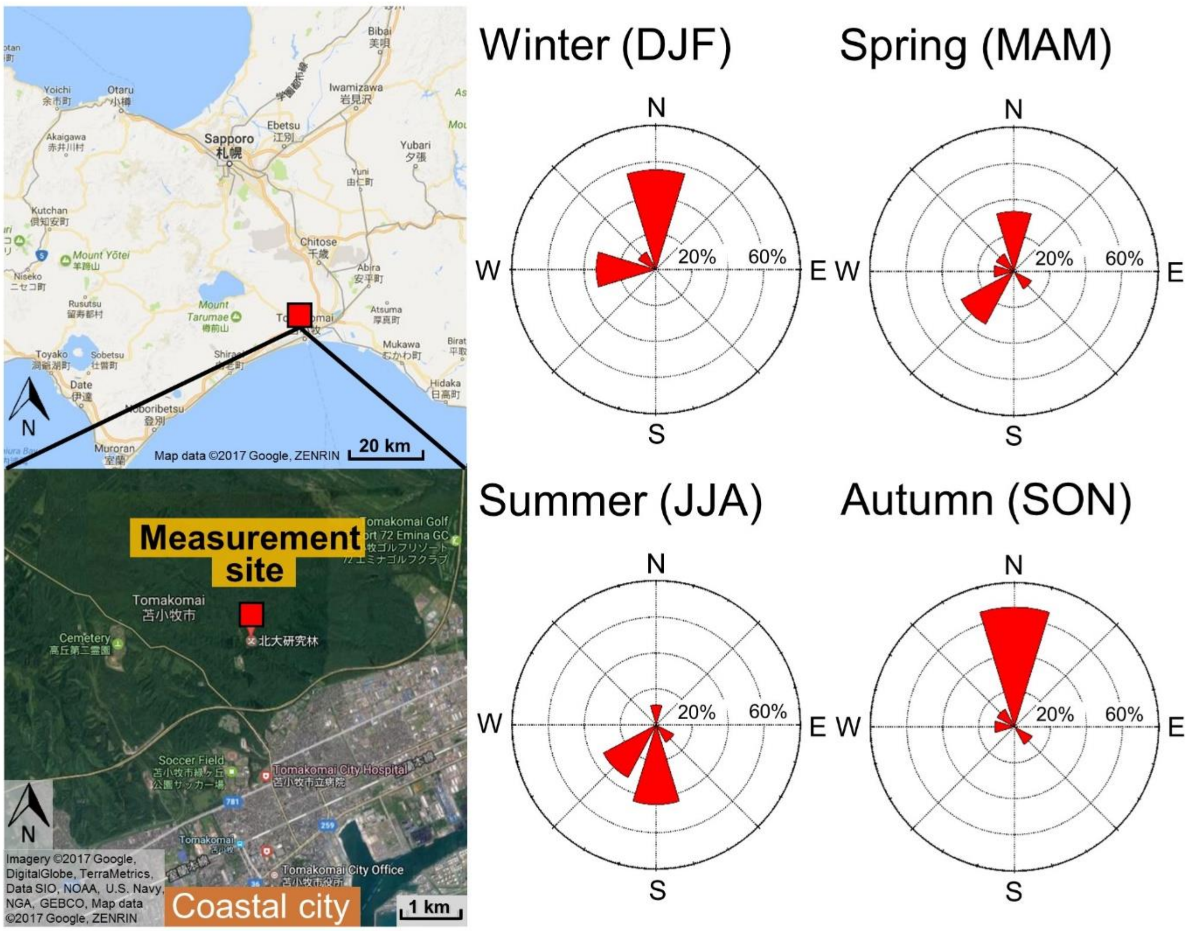

Figure S1. Location of the sampling site (Tomakomai Experimental Forest, TOEF) and the observed frequencies of local wind directions with wind speed >2 m s^{-1} for the year 2013. Map data ©2017 Google, ZENRIN; Imagery ©2017 Google, DigitalGlobe, TerraMetrics, Data SIO, NOAA, U.S. Navy, NGA, GEBCO (https://maps.google.com/). Maps are modified with Microsoft PowerPoint 2013.

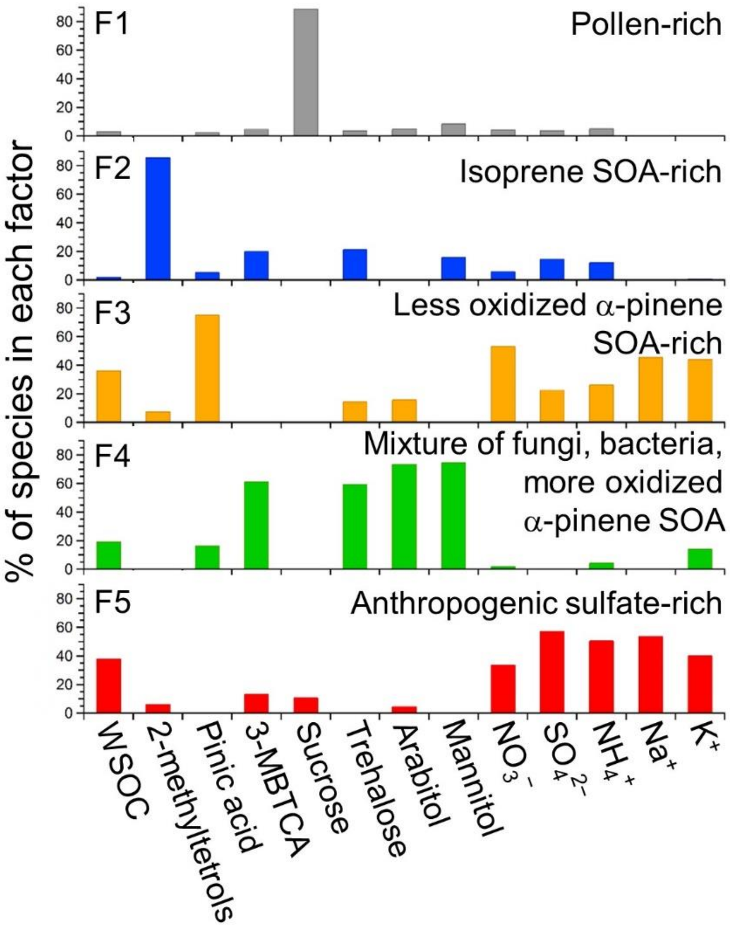

Figure S2. Five factor profiles derived from the PMF solution. The percentage of chemical species in each factor is shown.
